# Persistent Anemia Revealing Underlying B-cell Malignancies: A Report of Two Cases Highlighting a Diagnostic Gap in Primary Care

**DOI:** 10.7759/cureus.100645

**Published:** 2026-01-02

**Authors:** Chibuzo C Manafa, Nnamdi Okoli, Onyekachi Ekowo, Stephen Adilih, Ngozi Ogo

**Affiliations:** 1 Family Medicine, Queensland Medical Clinic, Calgary, CAN; 2 Orthopaedics, Musgrave Park Hospital, Belfast, GBR

**Keywords:** anemia, early diagnosis, free light chain assay, monoclonal gammopathy, multiple myeloma, primary care, serum protein electrophoresis, waldenström macroglobulinemia

## Abstract

Persistent anemia in older adults is commonly attributed to nutritional deficiency, chronic disease, or age-related change, particularly when routine laboratory investigations are unrevealing. However, plasma cell and lymphoplasmacytic disorders may present subtly, with anemia as the only consistent abnormality. We report two cases in which extended laboratory evaluation led to early identification of clinically significant B-cell malignancies.

The first case involved a man in his early 70s with stable, asymptomatic normocytic anemia despite normal iron studies, vitamin B12 and folate levels, renal function, thyroid studies, and liver enzymes. Serum protein electrophoresis revealed a monoclonal immunoglobulin G kappa paraprotein with an abnormal serum free light-chain ratio. Bone marrow biopsy confirmed clonal plasma cell infiltration consistent with smoldering multiple myeloma, and imaging demonstrated no myeloma-defining bone disease. The patient remains under active surveillance.

The second case involved a man in his late 70s with a several-year history of progressive macrocytic anemia and repeatedly normal first-line investigations. Extended testing revealed a marked immunoglobulin M lambda monoclonal gammopathy, and bone marrow biopsy confirmed Waldenström macroglobulinemia. He required treatment due to severe anemia and responded to bendamustine-rituximab therapy.

These cases highlight the diagnostic value of serum protein electrophoresis, quantitative immunoglobulins, and serum free light-chain testing in patients with persistent unexplained anemia. Early use of extended investigations can facilitate timely diagnosis before end-organ damage occurs and details the critical role of primary care clinicians in recognizing patterns that warrant escalation beyond routine anemia algorithms.

## Introduction

Anemia is commonly encountered in elderly patients, with a prevalence greater than 10% in this age group [1]. Primary care physicians are usually the first point of contact, and typical initial evaluations include a complete blood count, iron studies, vitamin B12 and folate levels, renal function, and thyroid testing [2]. When these investigations are negative, clinicians often attribute mild anemia to age-related changes or chronic disease, particularly in the absence of symptoms. While this approach is often pragmatic, it risks overlooking less common but clinically important conditions, such as monoclonal gammopathies and B-cell malignancies.

Multiple myeloma (MM) and Waldenström macroglobulinemia (WM) frequently present with nonspecific laboratory abnormalities, and anemia may be among the earliest detectable changes [3,4]. Classic manifestations, including bone pain, renal impairment, hypercalcemia, neuropathy, or hyperviscosity, tend to appear only after significant disease progression [4]. This creates a window in which early detection is possible, but may be missed if diagnostic evaluation ends after routine testing [5].

Serum protein electrophoresis (SPEP), quantitative immunoglobulin assays, and serum free light-chain (FLC) tests are readily available and informative [6]. However, these are not commonly incorporated into initial anemia algorithms, creating a diagnostic gap when routine tests appear normal. Although expert groups, including the International Myeloma Working Group, highlight the importance of SPEP in unexplained anemia, utilization in primary care remains variable [7,8].

The two cases presented here demonstrate how extended evaluation enabled early identification of MM and WM in asymptomatic individuals whose routine laboratory tests did not initially suggest a cause. They highlight the importance of considering monoclonal gammopathies when anemia persists despite normal first-line testing and reinforce the crucial role of primary-care physicians in recognizing laboratory patterns warranting extended investigation.

## Case presentation

Case 1: MM identified through extended evaluation of mild, persistent anemia

A male patient in his early 70s was noted during routine testing to have persistent normocytic anemia, with hemoglobin values ranging from 127 to 130 g/L over three months. He remained very active, reported no fatigue, weight loss, bone pain, or constitutional symptoms. Vital signs were normal, and his chest and abdominal examinations were normal. His hemoglobin pattern remained stable, and no clear etiology was noted.

Initial investigations showed hemoglobin of 127-130 g/L, mean corpuscular volume (MCV) 96-98 fL, normal ferritin, normal iron studies, normal vitamin B12 and folate, normal thyroid-stimulating hormone (TSH), normal creatinine and estimated glomerular filtration rate (eGFR), normal calcium, and normal liver enzymes. His reticulocyte count of 1.5 was appropriately low for the degree of anemia. There were no features to suggest marrow infiltration, chronic inflammatory disease, or hemolysis. In many cases, such mild anemia might be monitored without further investigation. However, given persistent unexplained anemia, extended laboratory testing was pursued. SPEP demonstrated a monoclonal IgG-kappa spike of 27.88 g/L. The FLC ratio was markedly abnormal at 6.01. Quantitative immunoglobulin testing showed elevated IgG with suppression of immunoglobulin M (IgM). Total protein was mildly elevated at 83 g/L. A chronological summary of hematologic, biochemical, and extended myeloma investigations is presented in Table 1.

**Table 1:** Chronological Laboratory Investigations in Case 1 (Smouldering Multiple Myeloma) Chronological summary of laboratory investigations performed during the evaluation of persistent normocytic anemia in Case 1. The table demonstrates repeatedly normal first-line anemia investigations followed by extended testing that identified a monoclonal gammopathy. Reference ranges are provided for all parameters. A hyphen (-) indicates tests not performed at that time point. Hb: hemoglobin; MCV: mean corpuscular volume; SPEP: serum protein electrophoresis; FLC: free light-chain; Ig: immunoglobulin; eGFR: estimated glomerular filtration rate

Parameter	Reference Range	August 2025	September 2025	November 2025
Hemoglobin (g/L)	135-175	127	129	130
MCV (fL)	80-100	98	96	97
Ferritin (µg/L)	30-500	367	-	-
Vitamin B12 (pmol/L)	>160	-	317	-
Folate (nmol/L)	>10	-	32.3	-
Thyroid-stimulating hormone (mIU/L)	0.4-4.0	1.85	1.76	-
Creatinine (µmol/L)	50-120	103	-	97
Estimated GFR (mL/min/1.73 m^2^)	≥60	62	-	66
Calcium (mmol/L)	2.10-2.60	2.34	-	2.45
Alanine aminotransferase (U/L)	<70	55	-	-
Reticulocyte count (%)	0.4-2.0	-	1.5	-
Total protein (g/L)	62-82	-	-	83
SPEP	-	-	-	IgG-κ monoclonal spike (27.88g/L)
Immunoglobulin G (g/L)	6.8-18.00	-	-	27.88
Immunoglobulin A (g/L)	0.6-4.20	-	-	0.78
Immunoglobulin M (g/L)	0.4-3.00	-	-	0.37
Free light-chain ratio (κ/λ)	0.26-1.65	-	-	6.01

**Figure 1:**
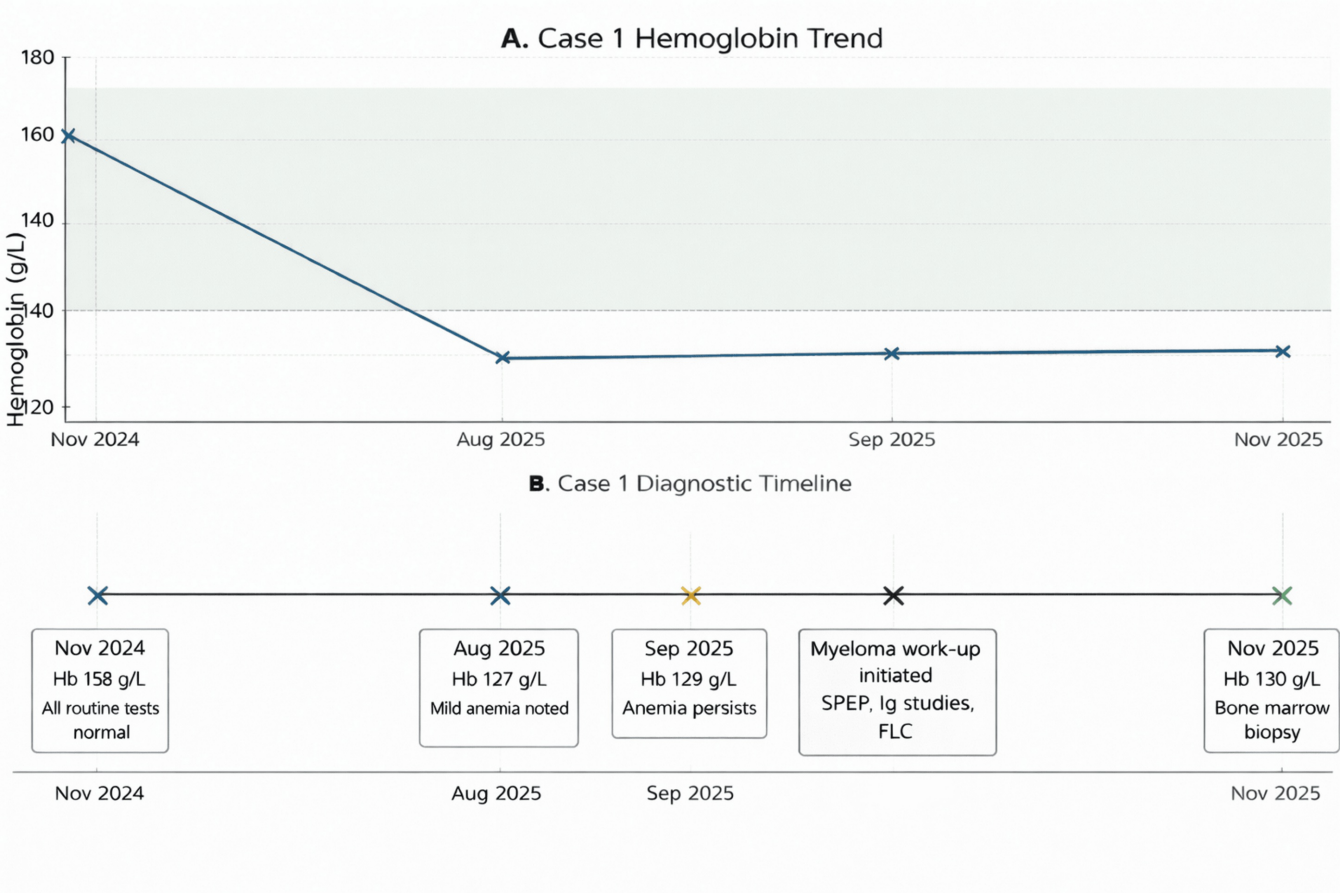
Hemoglobin Trend and Diagnostic Timeline in Case 1 (Smouldering Multiple Myeloma) (A) Serial hemoglobin measurements over time demonstrating persistent mild normocytic anemia preceding diagnosis. The shaded region represents the laboratory reference range for hemoglobin. (B) Diagnostic timeline illustrating key clinical milestones, including recognition of persistent anemia, ordering of SPEP, detection of an IgG-kappa monoclonal spike, and confirmation of a plasma cell disorder consistent with smoldering multiple myeloma on bone marrow biopsy. Hb: hemoglobin; SPEP: serum protein electrophoresis; FLC: free light-chain

Hematology referral was arranged, and bone marrow biopsy confirmed clonal plasma-cell infiltration meeting the ≥10% threshold, with kappa light-chain restriction. The patient had no myeloma-defining events, including CRAB (hypercalcemia, renal impairment, anemia, and bone lesions) features. Anemia was not severe (hemoglobin ≥100 g/L and <20 g/L below the lower limit of normal), and no definitive lytic bone lesions or myeloma-defining focal lesions were identified on imaging. He also had no SLiM biomarkers (≥60% clonal plasma cells, involved/uninvolved light-chain ratio ≥100, or ≥1 magnetic resonance imaging focal lesion ≥5 mm), consistent with smoldering MM. He remains under active surveillance. Without SPEP and immunoglobulin testing, diagnosis would likely have been delayed until organ involvement occurred. The evolution of hemoglobin values and key diagnostic milestones leading to the identification of smoldering MM are summarized in Figure 1.

Early identification allowed appropriate risk stratification using the revised International Staging System and initiation of active surveillance. The patient is currently under three-monthly monitoring of paraprotein, hemoglobin, renal function, and calcium, and remains asymptomatic.

Case 2: WM presenting as a longstanding macrocytic anemia

A male patient in his late 70s had a several-year history of mild macrocytosis and gradually progressive anemia. He reported no alcohol intake. Hemoglobin values were in the 140 g/L range in 2021, declining to the high 130s in 2022 and to 111 g/L in 2024, prompting referral to hematology. Further assessment was performed after subsequent anemia progression, with hemoglobin reaching 86 g/L in 2025. He denied B symptoms (fever, night sweats, and unintentional weight loss), neuropathy, visual disturbances, bleeding, fatigue, or constitutional complaints. He remained active and functionally well. Vital signs were normal, and chest and abdominal exams revealed no abnormalities.

**Figure 2:**
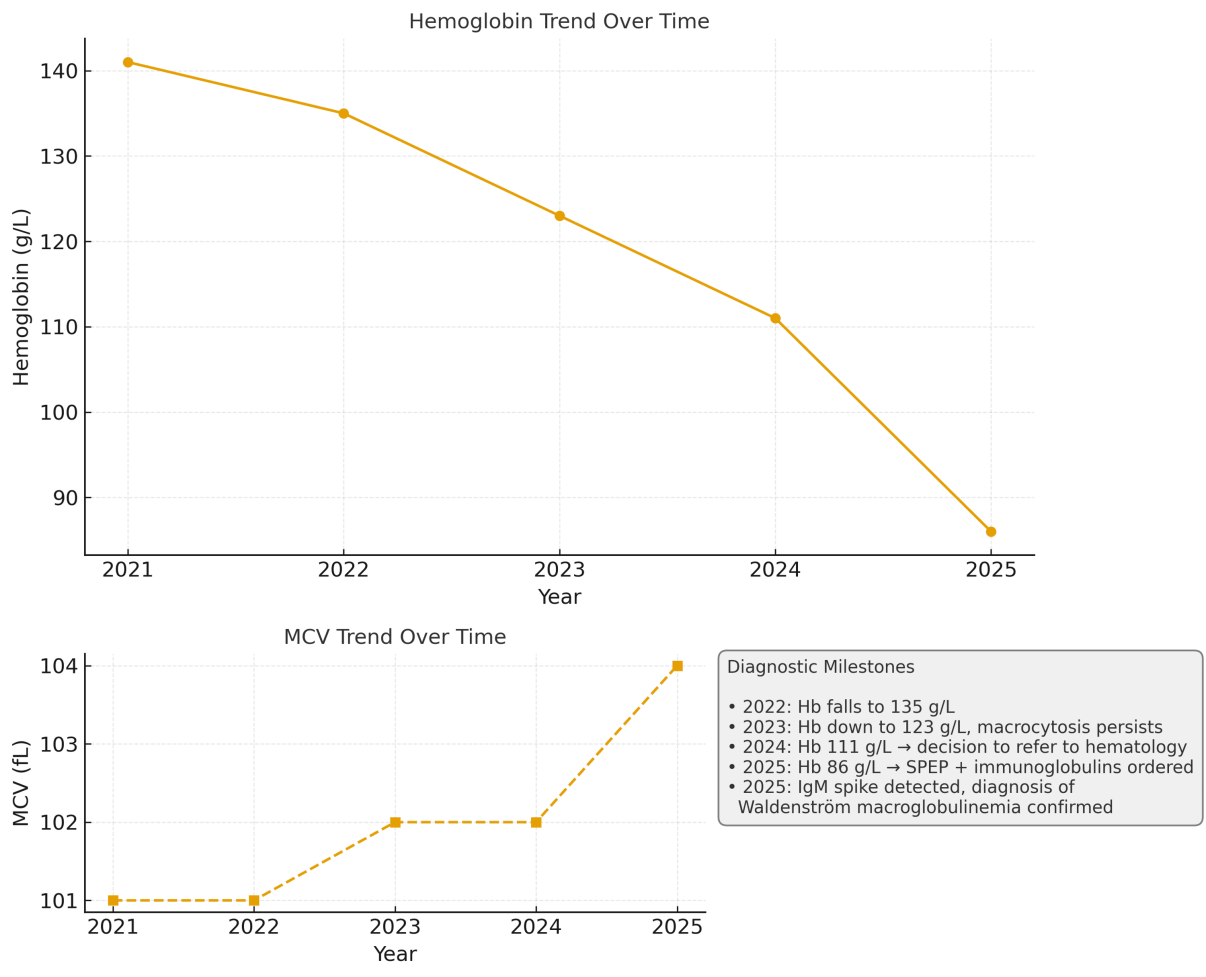
Hemoglobin and MCV Trends Over Time in Case 2 Hemoglobin and MCV trends over time in Case 2, showing progressive anemia with persistent macrocytosis preceding the diagnosis of Waldenström macroglobulinemia. Hb: hemoglobin; MCV: mean corpuscular volume; SPEP: serum protein electrophoresis

Investigations demonstrated normal ferritin levels, normal TSH, preserved renal and liver function, and no laboratory evidence suggestive of hemolysis. Vitamin B12 and folate levels, assessed during later evaluation, were within the normal range. SPEP revealed a prominent IgM-lambda monoclonal spike. Immunoglobulin testing confirmed IgM of 109 g/L, with suppression of immunoglobulin G (IgG) and immunoglobulin A (IgA). Serum FLC assays demonstrated elevated lambda light chains with an inverted kappa/lambda ratio. Baseline and extended laboratory investigations are summarized in Table 2. Serial hemoglobin and MCV trends over time, illustrating progressive macrocytic anemia preceding diagnosis, are shown in Figure 2.

**Table 2:** Chronological Laboratory Investigations in Case 2 (Waldenström Macroglobulinemia) Summary of laboratory investigations in Case 2 showing progressive macrocytic anemia with normal first-line studies and subsequent identification of an IgM-lambda monoclonal gammopathy on extended testing.

Parameter	Reference Range	September 2022	October 2023	September 2024	May 2025	August 2025	September 2025	December 2025
Hemoglobin (g/L)	135-175	135	123	111	114	86	102	121
Mean corpuscular volume (fL)	80-100	101	102	102	104	110	107	102
Ferritin (µg/L)	30-500	-	-	-	-	201	-	-
Vitamin B12 (pmol/L)	≥160	-	-	221	294	288	-	-
Folate (nmol/L)	≥10.0	-	-	23.9	-	15.8	-	-
Thyroid-stimulating hormone (mIU/L)	0.2-6.50	-	-	4.28	-	-	-	-
Alanine aminotransferase (U/L)	<60	12	16	15	-	12	12	16
Creatinine (µmol/L)	50-120	66	73	73	-	75	65	84
Total protein (g/L)	62-82	-	-	-	-	111	115	94
Immunoglobulin M (IgM) (g/L)	0.40-3.00	-	-	-	-	88.60	109.74	72.62
Immunoglobulin G (IgG) (g/L)	6.80-18.00	-	-	-	-	3.96	3.68	3.57
Immunoglobulin A (IgA) (g/L)	0.60-4.20	-	-	-	-	0.43	0.32	0.14
Kappa free light-chain (mg/L)	3.3-19.4	-	-	-	-	-	8.9	2.5
Lambda free light-chain (mg/L)	5.7-26.3	-	-	-	-	-	47.1	12.4
Kappa/lambda ratio	0.26-1.65	-	-	-	-	-	0.19	0.20
Lactate dehydrogenase (U/L)	120-250	-	-	-	-	127	142	137

Bone marrow biopsy showed lymphoplasmacytic infiltration greater than 10%, lambda light-chain restriction, and a borderline-positive myeloid differentiation primary response 88 (MYD88) L265P mutation, consistent with WM. Treatment was indicated based on severe anemia, high IgM burden, and progressive disease. Bendamustine-rituximab chemo-immunotherapy was initiated, resulting in an improvement in hemoglobin to 121 g/L after three cycles and a reduction in IgM to 72 g/L.

Investigations

Both patients underwent extensive first-line testing, including complete blood count, ferritin and iron studies, vitamin B12 and folate levels, renal and liver function tests, thyroid studies, calcium, and lactate dehydrogenase. Extended testing, which ultimately revealed the underlying diagnoses, included SPEP, quantitative immunoglobulins, serum FLCs, and total protein. Bone marrow aspiration and biopsy confirmed the underlying clonal disorders.

**Figure 3:**
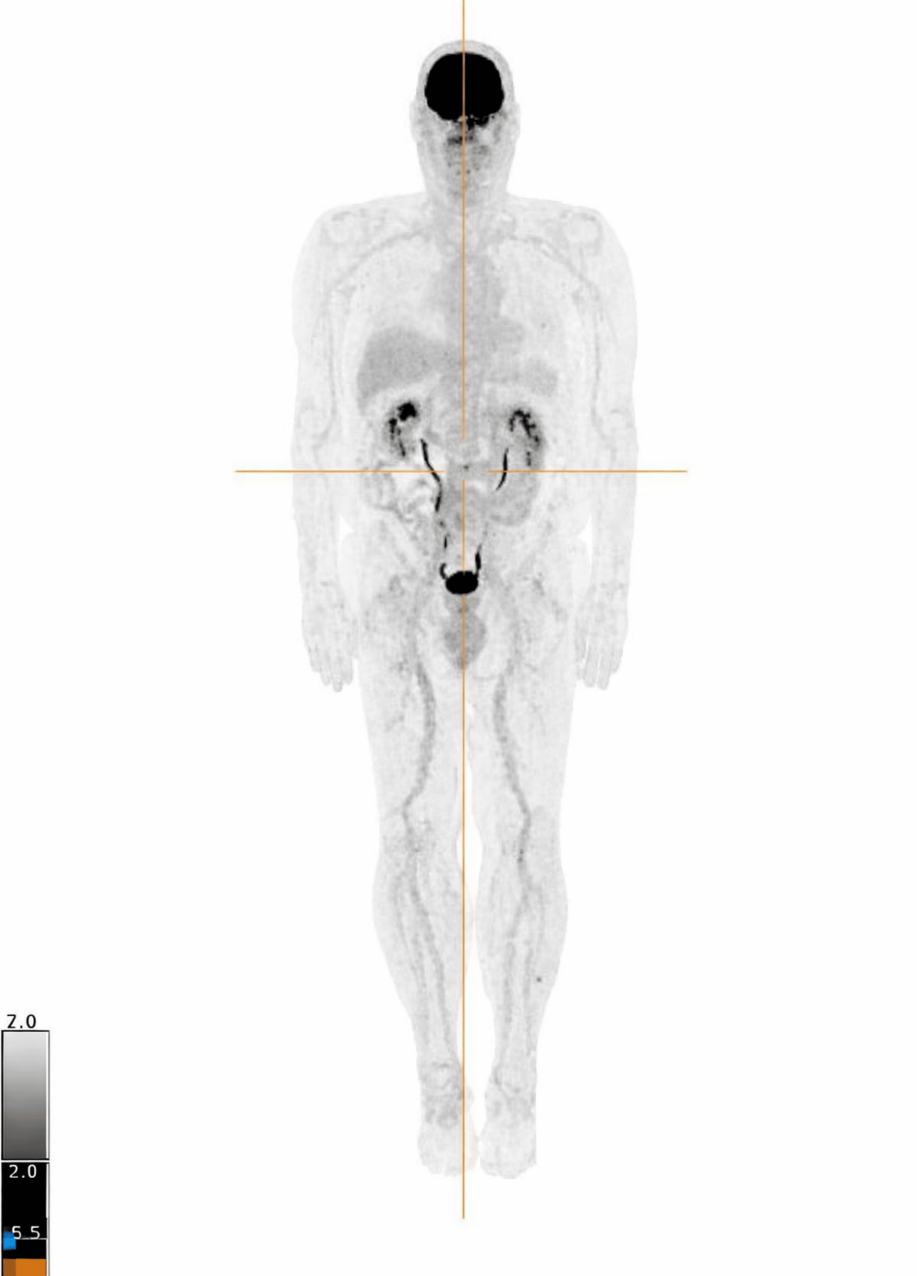
Whole-Body FDG PET/CT in Case 1 Maximum intensity projection image from a whole-body (18F)-fluorodeoxyglucose positron emission tomography/computed tomography (FDG PET/CT) scan demonstrating no evidence of widespread FDG-avid skeletal or extramedullary disease. A small focal area of moderate FDG uptake is seen at the anterior aspect of the L3 vertebral body (crosshairs), without definitive features of active myeloma. Findings were considered indeterminate and managed with active surveillance.

Imaging in Case 1 included a whole-body (18F)-fluorodeoxyglucose positron emission tomography/computed tomography (FDG PET/CT) scan, which demonstrated no widespread FDG-avid skeletal or extramedullary disease. A single small focus of moderate FDG uptake was noted in the anterior aspect of the L3 vertebral body, without definitive radiologic features of active myeloma. These findings were interpreted as indeterminate and managed with active surveillance (Figure 3).

Differential diagnosis

In Case 1, persistent normocytic anemia prompted consideration of anemia of chronic disease, but normal inflammatory markers and the absence of chronic illness excluded this. Myelodysplastic syndrome was considered less likely due to normal MCV and no dysplastic features on smear. Occult gastrointestinal bleeding was excluded by normal iron indices and normal fecal immunohistochemistry test (FIT). Normal TSH and eGFR excluded hypothyroidism and chronic kidney disease. Ultimately, monoclonal gammopathy became the most likely diagnosis, confirmed by SPEP and bone marrow biopsy.

In Case 2, progressive macrocytic anemia prompted evaluation for vitamin B12 or folate deficiency, both of which were repeatedly normal. Hypothyroidism, liver disease, alcohol excess, and medication-related macrocytosis were excluded. The combination of worsening anemia and macrocytosis led to evaluation for a clonal disorder, ultimately confirming WM.

Treatment

Case 1 was diagnosed as smoldering MM by hematology in the absence of CRAB features. He continues to have active surveillance with serial monitoring. No treatment has been initiated, and therapy will only begin if progression to symptomatic myeloma is confirmed.

Case 2 required treatment due to symptomatic anemia and high IgM burden. He has been enrolled for a six-cycle chemotherapy treatment using the bendamustine-rituximab regimen every 28 days. After two cycles, he has shown a partial response, with hemoglobin rising to 121 g/L and IgM falling to 72 g/L.

Outcome and follow-up

Case 1 remains under active surveillance and is currently asymptomatic with stable hemoglobin and paraprotein levels. Case 2 continues treatment with excellent tolerance and sustained improvement in hemoglobin and IgM levels. Both patients are co-managed by primary care and hematology, denoting the value of collaborative chronic disease management.

## Discussion

These two cases showcase the importance of maintaining a broad differential diagnosis in older adults presenting with anemia, particularly when first-line investigations fail to reveal a cause. While nutritional deficiencies, chronic kidney disease, and inflammatory states are frequent culprits, less common disorders such as MM and WM may initially present only with mild anemia or macrocytosis [1,4].

Current guidelines emphasize a stepwise approach focused on common causes [2]. However, this approach may fail to detect early plasma-cell or lymphoplasmacytic disorders. Up to 12% of patients with unexplained anemia may harbor a monoclonal gammopathy, yet SPEP testing remains low in primary care [9]. These cases illustrate how clinically significant disorders may remain undetected for long periods unless extended testing is pursued.

SPEP is a simple test that separates serum proteins into distinct fractions, enabling the detection of monoclonal proteins indicative of clonal plasma cell or lymphocyte proliferation [10]. Performing immunofixation electrophoresis alongside SPEP increases the specificity and sensitivity by confirming and typing the paraprotein. In addition, adding serum-FLC assays further improves diagnostic accuracy, especially in light-chain-only myeloma [11]. Documented barriers include limited awareness, the possibility of false-positive results, and uncertainty about when the test is relevant in primary care [12]. In persistent unexplained anemia, the pretest probability of a clonal process supports the use of these tests [8].

Extended testing is particularly appropriate when first-line investigations do not reveal the etiology of anemia, when unexplained macrocytosis is present, when the decline in hemoglobin is progressive, or when patients over 60 develop new-onset anemia.

With early detection, patients can be easily stratified by risk and prognosis. There is also an opportunity to monitor disease progression, prevent complications such as renal failure or hyperviscosity, and improve access to clinical trials [13]. However, early diagnosis may also cause anxiety, increase healthcare costs, and identify benign monoclonal gammopathy of undetermined significance (MGUS), which is common and often non-progressive, particularly in older adults [14].

Although both conditions described here are B-cell malignancies characterized by monoclonal protein production, they differ significantly in immunoglobulin type, mutation profile, clinical phenotype, and prognosis [14]. A comparison of key pathological and clinical features that distinguish MM from WM, which informed diagnostic and management decisions in these cases, is presented in Table 3. Given the continuity and comprehensiveness of primary care, general practitioners are well-positioned to detect early or evolving abnormalities, initiate appropriate investigations, and coordinate timely referral when needed [15].

**Table 3:** Key Distinguishing Features Between MM and WM Relevant to Primary Care Comparison of major pathological, clinical, and therapeutic features distinguishing MM and WM [8,14,16]. Ig: immunoglobulin; CRAB: hypercalcemia, renal impairment, anemia, bone lesions; MYD88: myeloid differentiation primary response 88

Feature	Multiple Myeloma (MM)	Waldenström Macroglobulinemia (WM)
Cell of origin	Clonal plasma cell proliferation	Lymphoplasmacytic lymphoma with plasmacytic differentiation
Paraprotein type	Usually IgG or IgA (Case 1: IgG-κ)	IgM monoclonal protein
Genetics	Various cytogenetic abnormalities; no single hallmark mutation	MYD88 L265P mutation (~90%) [16]
Typical clinical features	CRAB features: Hypercalcemia, renal impairment, anemia, bone lesions (lytic lesions are common)	Symptoms due to IgM hyperviscosity or marrow/lymphoid involvement: Hyperviscosity, peripheral neuropathy, cytopenias, lymphadenopathy/splenomegaly
Bone disease	Common - lytic bone lesions	Rare - bone involvement unusual
When to treat	Treat when symptomatic. High-risk smouldering MM may be considered for early therapy in selected patients/clinical trials.	Treat when symptomatic (anemia, hyperviscosity, neuropathy, organomegaly, etc.)
Survival	Median survival >10 years with novel agents	Median survival 8-10 years; many have had indolent disease for decades

Future work should focus on optimizing testing pathways, assessing the cost-effectiveness of extended testing, and evaluating the impact of early diagnosis on patients.

## Conclusions

These cases highlight the importance of considering monoclonal gammopathies when anemia persists despite normal first-line testing. By extending the evaluations with SPEP and immunoglobulin panels, some silent but severe hematologic malignancies can be identified. Primary care clinicians, through longitudinal monitoring, play a critical role in early recognition.
